# Validity of distress thermometer for caregivers of cancer patients in Saudi Arabia

**DOI:** 10.1371/journal.pone.0344441

**Published:** 2026-03-12

**Authors:** Muneera A. AlKhorayef, Manal M. AlJurbua, Nouf A. AlHussaini, Reema A. AlMasoud, Ajeed M. Al Rashoud, Abdulillah M. Al Rashoud, Abdulmajeed A. Zarbah, Fahad D. Alosaimi

**Affiliations:** 1 Mental Health Department, King Fahad Medical City, Second Health Cluster, Riyadh, Saudi Arabia; 2 Psychiatry Department, King Saud University Medical City, Riyadh, Saudi Arabia; 3 Family Medicine Department, College of Medicine, King Saud University, Riyadh, Saudi Arabia; 4 Family Medicine Department, King Saud Medical City, First Health Cluster, Riyadh, Saudi Arabia; 5 Internal Medicine Department, King Fahad Medical City, Second Health Cluster, Riyadh, Saudi Arabia; 6 College of Medicine, King Saud University, Riyadh, Saudi Arabia; 7 Psychiatry Department, College of Medicine, Imam Abdulrahman Bin Faisal University, Dammam, Saudi Arabia; 8 Psychiatry Department, College of Medicine, King Saud University, Riyadh, Saudi Arabia; King Fahd Military Medical Complex, SAUDI ARABIA

## Abstract

**Background:**

Caregivers of cancer patients often face high levels of psychological distress, yet routine screening in clinical settings remains limited. The Distress Thermometer (DT) offers a brief and practical tool for identifying distress; however, its use among caregivers in Saudi Arabia has not been well studied. This study aims to validate the Arabic version of the DT in this population, determine its optimal cutoff scores, and explore associations with anxiety, depression, and demographic factors.

**Methods:**

A cross-sectional study was conducted at King Saud University Medical City (KSUMC), enrolling 275 caregivers. Participants completed the Arabic versions of the DT and the Hospital Anxiety and Depression Scale (HADS). Receiver Operating Characteristic (ROC) curve analysis was used to assess the DT’s predictive validity for anxiety and depression, while multivariable regression identified predictors of distress.

**Results:**

Clinically significant distress (DT ≥ 5) was reported **by** 44% of caregivers. Anxiety symptoms were present in 24%, and depression in 25%. DT scores were strongly correlated with HADS total (ρ = 0.618), HADS-anxiety (ρ = 0.537), and HADS-depression (ρ = 0.562) scores (all p < 0.001). The DT demonstrated excellent predictive validity, with an area under the curve (AUC) of 0.816 for anxiety and 0.818 for depression. The optimal cutoff scores were ≥5.5 and ≥6.5 for anxiety and depression, respectively. Female caregivers and those supporting patients with advanced-stage cancer reported significantly higher distress.

**Conclusion:**

The Arabic version of the DT is a valid and practical tool for identifying psychological distress among cancer caregivers in Saudi Arabia. Its brevity makes it suitable for routine clinical use. Further research is necessary to develop effective distress screening and intervention programs, particularly for caregivers of patients with advanced cancer, to ensure timely psychological support.

## Introduction

The global burden of cancer is expected to rise in the coming years as a result of population aging and growth [1]. This trend is particularly evident in Saudi Arabia, which is experiencing an increased prevalence of cancer compared to Western nations [1]. It is even predicted that by 2030 the cancer burden in the country will increase by five- to tenfold due to the population’s adoption of a Western lifestyle [2].

A cancer diagnosis represents a critical turning point not only for patients but also for their families [1]. In Saudi Arabia, where the family system is the primary social structure, relatives are chiefly responsible for providing care for those with chronic illnesses [3]. These caregivers, who often provide unpaid, demanding care that is physically, emotionally, and financially burdensome [4], are integral to successful cancer management and are routinely involved by physicians in treatment planning and decision-making [3].

However, this crucial role comes at a significant cost. Caregivers frequently face profound psychological distress, with its prevalence ranging from 15% to 50%, which is as common among them as it is among patients themselves [5]. This impact is often greater on family members than on patients [1], manifesting as anxiety (affecting 33% of caregivers versus 25% of patients) and worries about meeting the patient’s needs, decision-making, and balancing their own lives [6,7]. Over time, the immense emotional and physical costs of caregiving can lead to a quality of life that mirrors the suffering of the cancer patient [3,6]. Studying their psychological well-being is therefore crucial for providing necessary support, which in turn benefits both caregiver well-being and the quality of patient care [2].

Addressing this distress is critical, as screening and intervention are linked to better quality of life for caregivers and potentially improved treatment outcomes for patients [4]. While validated tools like the HADS and Zarit Burden Interview (ZBI) exist in Arabic for Saudi caregivers [2,8], they are often too lengthy or narrowly focused for practical use in busy clinical workflows [9,10].

To overcome these limitations, our study employed the Arabic version of the DT. This single-item, visual analogue scale provides a brief, valid, and non-invasive method to screen for distress “in the past week” on a scale from 0 (no distress) to 10 (extreme distress) [9,11]. We have previously validated the DT for cancer patients in Saudi Arabia, establishing a clinical cutoff score of 4 [11]. Although the DT has been used to study caregivers in other countries [5,12], no research has focused on this population within Saudi Arabia.

The DT is particularly well suited for caregiver populations because it captures global emotional distress without requiring lengthy diagnostic instruments [13].

Evidence shows that the DT demonstrates good psychometric properties and clinical utility not only in oncology caregivers but also in other caregiver groups, such as those supporting persons with multiple sclerosis [14].

Additionally, adapted versions of the DT, such as the DT-P for parents of chronically ill children and caregiver-specific DT-C versions for caregivers of children/adolescents with schizophrenia, have shown validity and reliability when compared with established measures of anxiety, depression, and parenting stress [15,16].

The DT has also been translated and validated across multiple languages and cultural contexts, reinforcing its flexibility and cross-cultural applicability as a brief distress screening tool [17].

In Saudi Arabia, widespread mental health stigma and prevailing cultural beliefs act as barriers to acknowledging psychological distress and accessing formal support [18], which underscores the importance of using brief, non-stigmatizing screening tools like the DT to identify distress in caregivers.

This rationale strengthens the choice of the DT for our study by highlighting its brevity, reliability, cultural adaptability, and suitability for detecting psychological distress in caregivers who may otherwise underreport symptoms due to stigma or social norms.

## Methodology

### Study design and setting

This cross-sectional study assessed the validity of the DT among caregivers of cancer patients at KSUMC in Riyadh, Saudi Arabia. Over a one-year period (6 April 2023–6 April 2024), we recruited caregivers from both inpatient wards and outpatient clinics. The study protocol received approval from the Institutional Review Board of the Faculty of Medicine at King Saud University (No. E-23–7770). All participants provided written informed consent prior to participation.

### Questionnaire development and pilot testing

The study questionnaire underwent initial review by experts in oncology and psychiatry; their feedback was discussed and incorporated to finalize the content. We then conducted a pilot test with 20 participants to assess reliability. Based on their feedback, specific questions were reworded for clarity before the final version was implemented.

### Data collection

Data collection was conducted by the authors, who received training on how to properly administer the questionnaire and record responses. This approach was taken to prevent potential misunderstandings if participants had filled it out themselves. Following the survey, clinical data were extracted from the patients’ medical records.

### Participants, recruitment, and sample size

Assuming that 50% of caregivers accompanying cancer patients would experience high levels of distress, and considering a finite population of approximately 700 caregivers, the minimum required sample size was estimated at 249 caregivers. This calculation was based on estimating the proportion of caregivers with distress with a 5% margin of error and a 95% confidence level. A total of 275 caregivers were enrolled using a convenience sampling strategy. Caregivers present in the hospital were invited directly, while those absent were contacted via telephone to complete the questionnaire. Non-participation occurred due to various reasons: some patients refused to involve their caregivers in the study, some patients did not have a primary caregiver, and some caregivers did not respond to the invitation.

**Fig 1:**
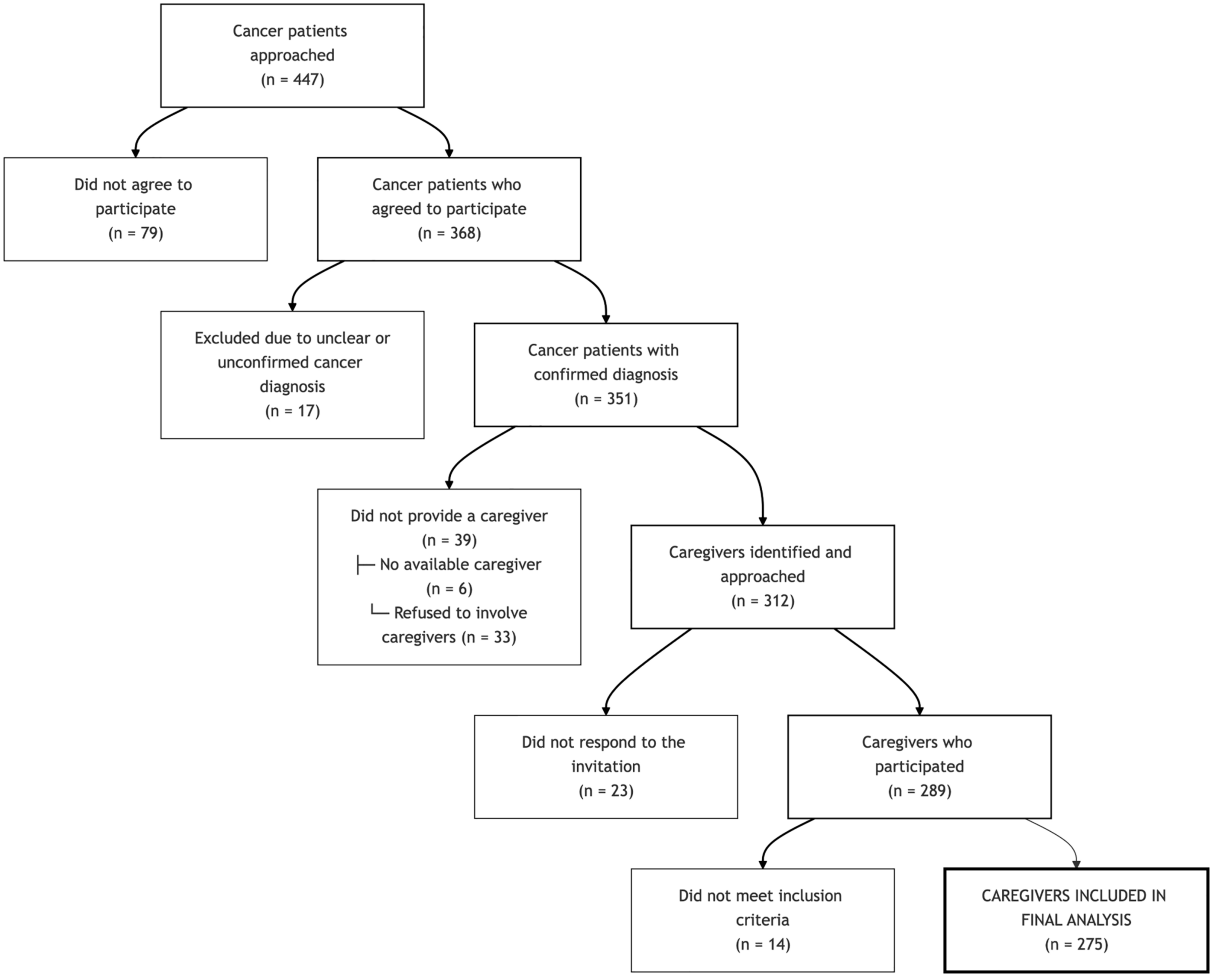
Flowchart of patient and caregiver recruitment.

Inclusion criteria required a definitive cancer diagnosis for the patient and an age of 18 years or older for both the patient and their self-identified primary caregiver. Exclusion criteria were a non-definitive cancer diagnosis or if either the patient or caregiver was under 18 years of age. Participant recruitment and inclusion are summarized in Fig 1.

### Translation and cultural adaptation of DT

The Arabic version of the DT used in this study was adopted from a previously validated translation developed for Saudi cancer patients by Alosaimi et al. [11]. The original translation followed a standardized cross-cultural adaptation process, including forward translation into Arabic, back-translation into English, and expert panel review to ensure semantic, conceptual, and cultural equivalence. The translated instrument was pilot-tested in the target population to assess clarity, acceptability, and cultural relevance, after which the final Arabic version was approved for clinical and research use.

### Measurements

**Distress thermometer (DT):** A previously validated Arabic version of the DT was used [11]. DT is a single-item, self-report measure that offers a brief, visual, and non-invasive alternative to longer, more cumbersome psychometric tests [13]. The patient marks the scale to answer, “How distressed have you been over the past week on a scale from 0 to 10?” [19] Its brevity and effectiveness have led to its recommendation by the National Comprehensive Cancer Network (NCCN) in their Clinical Practice Guidelines for identifying and managing distress. We proposed that a score of 5 or higher in our study indicates clinically significant distress [5,12]

**Hospital anxiety and depression scale (HADS):** a previously validated Arabic version was used in this study [20] The HADS questionnaire is commonly used to assess psychological distress among caregivers of cancer patients and has frequently been employed in studies validating the DT [12].

This 14-item questionnaire comprises two 7-item subscales measuring anxiety and depression. Participants rate their experience over the past week on a 4-point response scale (scored 0–3). Each subscale has a maximum score of 21 with a combined total score of up to 42 [21]. A score of ≤7 indicates non-anxiety/depression, 8 − 10 indicates borderline anxiety/depression, and 11 − 21 indicates definite anxiety/depression [20]. Thus, based on this previous research, we adopted a threshold subscale score of 8 for anxiety and depression in this study.

### Statistical analysis

Statistical analysis was performed using IBM SPSS Statistics version 29. Descriptive statistics, including mean and standard deviation or median and interquartile range (IQR), were used to summarize continuous variables depending on normality, while frequencies and percentages were used for categorical variables. The Kolmogorov-Smirnov test and histograms assessed normality, and multicollinearity was checked using Variance Inflation Factor (VIF) and Tolerance (Ti) indices. Spearman’s Rho correlation was used to evaluate relationships between variables, and the AUC-ROC was applied to assess the predictive validity of DT in identifying caregiver distress. Cronbach’s alpha tested the internal consistency of the DT and HADS.

To account for skewed data and overdispersion in outcomes such as distress, anxiety, and depression, a multivariable Generalized Linear Model (GLM) regression analysis with Gamma distribution was used. Risk Ratios (RR) with 95% confidence intervals identified predictors of caregiver outcomes. Statistical significance was set at an alpha level of 0.05. This comprehensive approach ensured that the findings regarding the validity and feasibility of the DT in caregiver populations were both robust and reliable.

## Results

### Socio-demographic characteristics of caregivers and cancer patients

The study included 275 cancer patient caregivers with a mean age of 39.59 years (SD = 12.44). Most caregivers were female (55.3%) and married (60.7%), and 571% held a university degree. Household income distribution showed that 26.9% earned between 5,000– < 10,000 SAR per month. In terms of family relationships, the majority of caregivers (55.3%) were the patients’ children, and most (73.1%) lived in the same household. The average caregiving duration was 2.19 years (SD = 3.21).

In terms of type of care provided: 95.6% assisted with daily living activities, and 91.6% provided emotional support or decision-making assistance, as shown in **Table 1**.

**Table 1:** Caregivers characteristics (N = 275).

Variable	N (%)	Mean (SD)
**Sex**		
Female	152 (55.3)	
Male	123 (44.7)	
**Age (years)**		39.59 (12.44)
**Age Group**		
18–23 years	22 (8)	
24–34 years	86 (31.3)	
35–44 years	77 (28)	
45–54 years	54 (19.6)	
55–64 years	27 (9.8)	
≥65 years	9 (3.3)	
**Marital Status**		
Single	93 (33.8)	
Widowed/Divorced	15 (5.5)	
Married	167 (60.7)	
**Educational Level**		
Intermediate or less	37 (13.5)	
High school degree	58 (21.1)	
University degree	157 (57.1)	
Post-graduate degree	23 (8.4)	
**Employment Status**		
Unemployed	81 (29.5)	
Student	26 (9.5)	
Retired	22 (8)	
Employed	146 (53.1)	
**Monthly Household Income (SAR)**		
<5,000 SAR	54 (19.6)	
5,000–9,999 SAR	74 (26.9)	
10,000–14,999 SAR	67 (24.4)	
15,000–19,999 SAR	37 (13.5)	
≥20,000 SAR	43 (15.6)	
**Number of children**		4.1 (2.21)
**Comorbidity**		
No	209 (76)	
Yes	66 (24)	
**Mental illness**		
No	268 (97.5)	
Yes	7 (2.5)	
**Duration as a Caregiver (years)**		2.19 (3.21)
<1 year	119 (43.3)	
1-2 years	89 (32.4)	
3-8 years	50 (18.2)	
≥9 years	17 (6.2)	
**Relationship to patient**		
Child	152 (55.3)	
Friend/Relative	9 (3.3)	
Parent	14 (5.1)	
Private Duty Nurse	1 (0.4)	
Sibling	44 (16)	
Spouse	55 (20)	
**Lives With Patient**		
No	74 (26.9)	
Yes	201 (73.1)	
**Type of Care Provided**		
Personal Care (feeding, cleaning, toileting)	122 (44.4)	
Daily Life Activities (transportation, medication)	263 (95.6)	
Other (emotional support, decision-making)	252 (91.6)	

Among the cancer patients, 74.5% were female. The most common diagnoses were breast cancer (40%) and gastrointestinal cancer (24.4%). Disease progression was advanced in most cases, with 30.9% at stage III and 37.8% at stage IV. Additionally, 47.6% of patients had comorbidities, and 9.1% had been diagnosed with mental illness—primarily depression (60%) and anxiety (20%), as summarized in **Table 2**.

**Table 2:** Patients characteristics (N = 275).

Variable	N (%)	Mean (SD)
**Sex**		
Female	205 (74.5)	
Male	70 (25.5)	
**Age (years)**		56.26 (12.9)
**Diagnosis**		
Breast	110 (40)	
Gastrointestinal	67 (24.4)	
Genitourinary	28 (10.2)	
Head and Neck	17 (6.2)	
Hematological	33 (12)	
Lung	10 (3.6)	
Malignant Peripheral Nerve Sheath	1 (0.4)	
Musculoskeletal	5 (1.8)	
Oral	1 (0.4)	
Skin	3 (1.1)	
**Disease Stage**		
Stage 0	10 (3.6)	
Stage 1	21 (7.6)	
Stage 2	55 (20)	
Stage 3	85 (30.9)	
Stage 4	104 (37.8)	
**Comorbidity**		
No	144 (52.4)	
Yes	131 (47.6)	
**Mental Illness**		
No	250 (90)	
Yes	25 (9.1)	

### Prevalence and correlation of distress, anxiety, and depression

The study revealed significant psychological burden among caregivers, with distress levels showing a mean score of 3.6 (SD = 2.79) on the DT. Notably, 44% of caregivers reported clinically significant distress levels (DT ≥ 5/10). Anxiety symptoms were particularly prevalent, with 12% of caregivers experiencing mild anxiety, 9.5% moderate anxiety, and 2.5% severe anxiety as measured by the HADS. Depression symptoms were also notable, affecting 14.9% of caregivers at mild levels, 8% at moderate levels, and 1.8% at severe levels (Table 3).

**Table 3:** Assessment of psychological factors among caregivers of cancer patients (N = 275).

Variable	N (%)	Mean (SD)
**Distress Thermometer (DT) Score**		3.6 (2.79)
DT ≤ 4 (Low distress)	154 (56) 121 (44)	
DT ≥ 5 (High distress)		
**Hospital anxiety and depression scale–anxiety subscale (HADS-A)**		5.05 (4.29)
0-7 points (Normal)	209 (76)	
8-10 points (Mild)	33 (12)	
11-15 points (Moderate)	26 (9.5)	
16-21 points (Severe)	7 (2.5)	
**Hospital anxiety and depression scale–depression subscale (HADS-D)**		5.4 (3.79)
0-7 points (Normal)	207 (75.3)	
8-10 points (Mild)	41 (14.9)	
11-15 points (Moderate)	22 (8)	
16-21 points (Severe)	5 (1.8)	
**HADS Total Score**		10.45 (7.17)

### Efficacy of DT in identifying caregiver anxiety and depression

**Fig 2:**
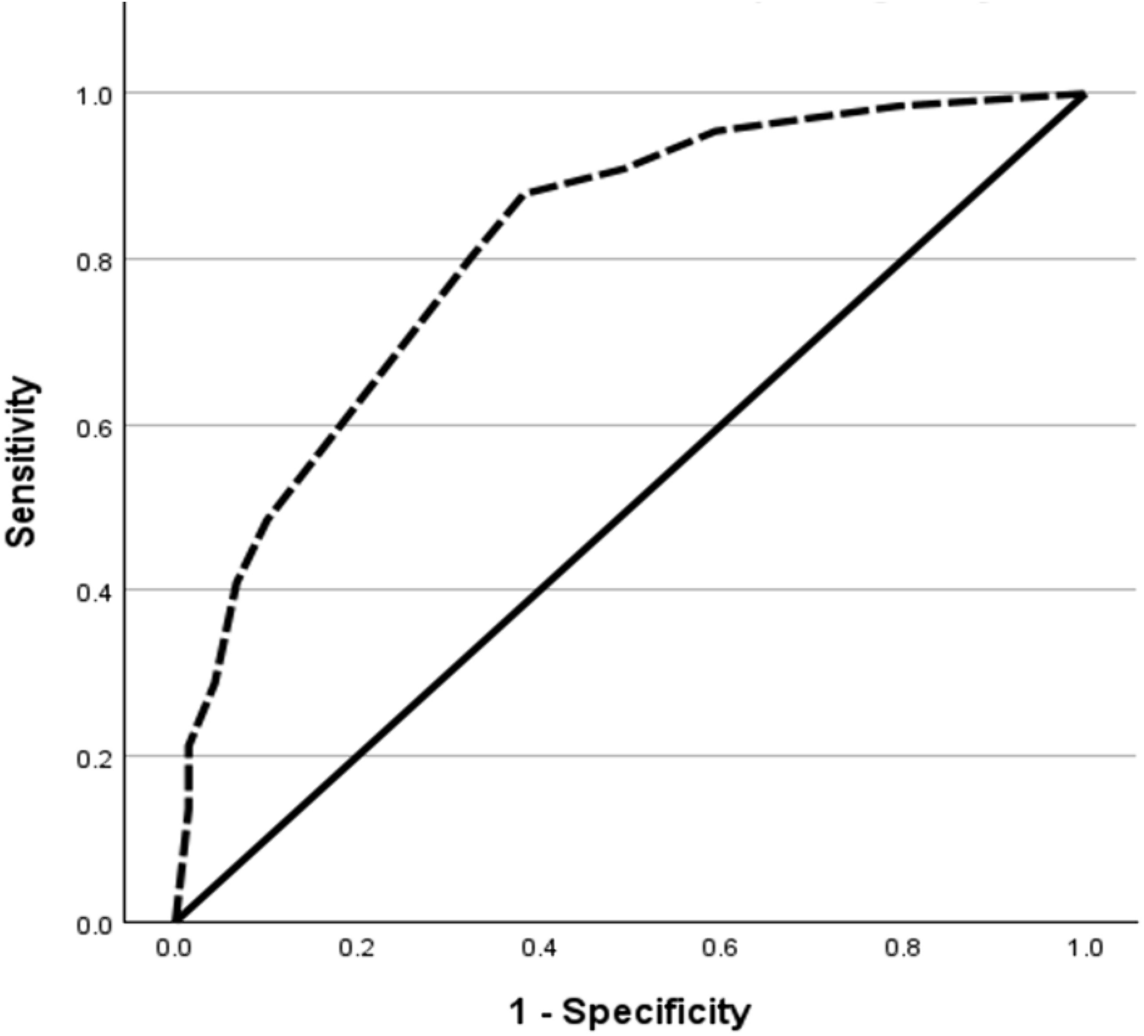
ROC curve of DT for predicting anxiety (HADS-A ≥ 8).

**Fig 3:**
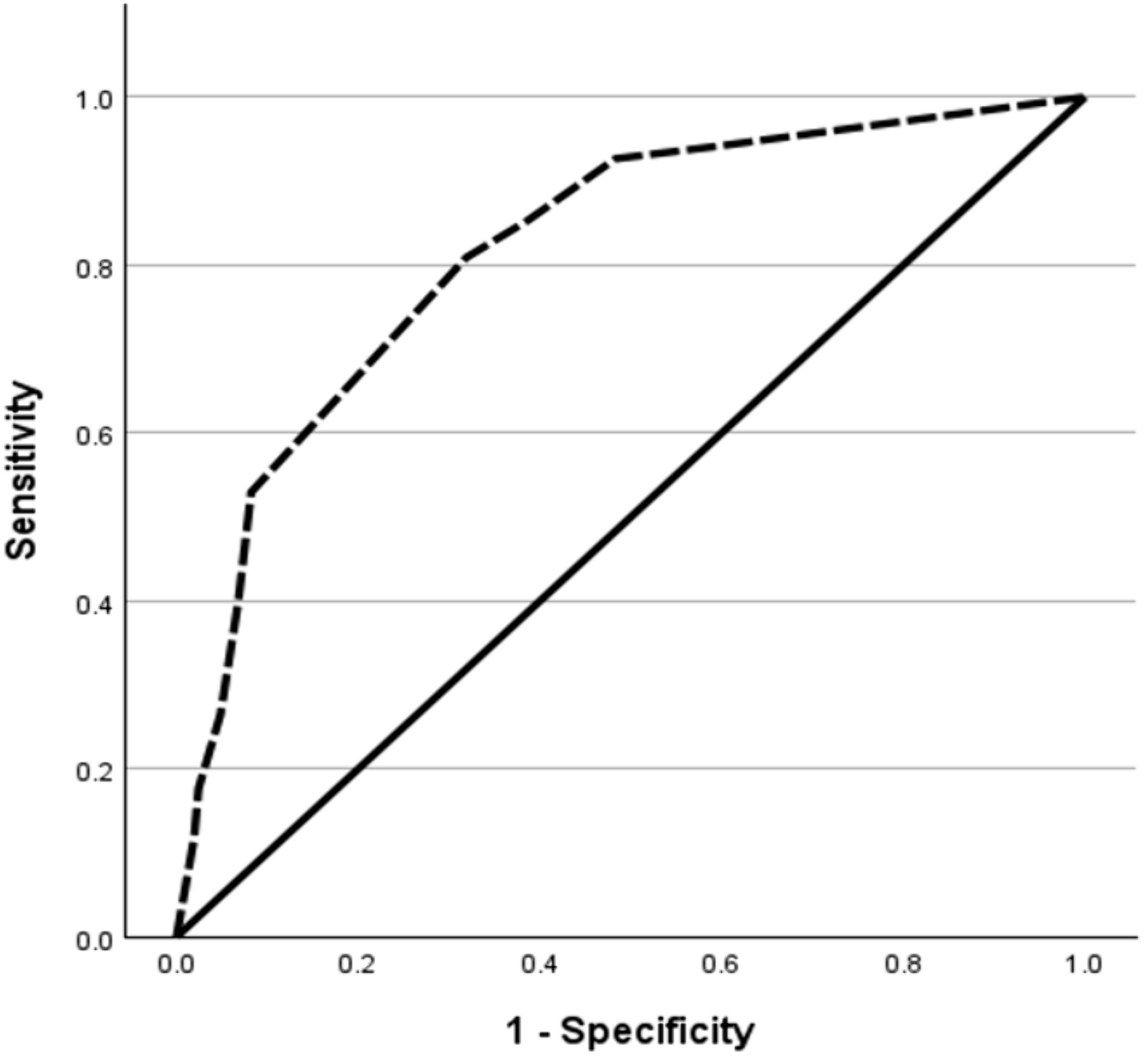
ROC curve of DT for predicting depression (HADS-D ≥ 8).

The DT demonstrated strong predictive validity for caregiver anxiety and depression, as shown by ROC curve analysis. For caregiver anxiety (measured by the HADS-A subscale), the DT showed excellent discrimination (AUC = 0.816, p < 0.001), with an optimal cutoff score of ≥5.5, identified by Youden Index analysis (sensitivity = 0.803, specificity = 0.60) The DT similarly predicted caregiver depression (HADS-D subscale) with high accuracy (AUC = 0.818, p < 0.001), using a slightly higher optimal cutoff of ≥6.5 (sensitivity = 0.54, specificity = 0.912). These results indicate that this brief single-item tool effectively identifies caregivers at risk for clinically significant anxiety and depression (Table 4; Figs 2 and 3).

**Table 4:** ROC analysis of DT predicting anxiety and depression (score ≥8).

Test Result Variable(s)	Area under the ROC	Lower 95% CI	Upper 95% CI	Youden Index	Cut off value	Sensitivity	Specificity	p-value
DT score predicting Anxiety (HADS-A ≥ 8)	0.816	0.759	0.872	0.342	5.500	0.803	0.60	<0.001
DT score predicting Depression (HADS-D ≥ 8)	0.818	0.761	0.876	0.447	6.500	0.54	0.912	<0.001

### Factors influencing caregiver distress levels

**Bivariate Spearman’s correlation analysis** demonstrated a strong and significant positive association between caregivers’ total HADS scores and their DT scores (Rho = 0.618, p < 0.001). Both HADS-A (Rho = 0.537, p < 0.001) and HADS-D (Rho = 0.562, p < 0.01) subscale scores were significantly and positively correlated with DT scores. In addition, caregivers’ DT scores showed a significant positive correlation with patients’ DT scores (Rho = 0.155, p < 0.05), as shown in Table 5.

**Table 5:** Bivariate spearman’s rho correlations between cancer patients’ and caregivers’ perceptions.

Caregiver variable	DT Score	HADS Total Score	HADS-A Score	HADS-D score	Duration as a caregiver	Caregiver Number of Children	Caregiver Age	Patient Age
Caregiver DT score	1.000							
Caregiver HADS total score	.618**							
Caregiver HADS-A score	.537**	.901**						
Caregiver HADS-D score	.562**	.871**	.573**					
Duration as a caregiver	0.023	0.003	−0.034	0.044				
Caregiver number of children	0.085	.184*	0.125	.209*	.212**			
Caregiver age	0.080	−0.018	−0.110	0.091	0.115	.496**		
Patients age	0.042	−0.025	−0.057	0.018	.153*	0.031	0.023	
Patients DT score	.155*	0.082	0.057	0.091	0.106	0.079	0.014	−0.039

**Correlation is significant at the 0.01 level (2-tailed). *Correlation is significant at the 0.05 level (2-tailed).

**Multivariable generalized linear regression analysis** revealed several key factors associated with caregivers’ perceived distress levels. Male caregivers reported significantly lower distress (17.9% less) than females (p = 0.006), while caregiver age showed no significant association (p = 0.466). Both caregiver HADS-A (RR = 1.039, p < 0.001) and HADS-D (RR = 1.062, p < 0.001) scores showed significant positive associations with DT scores, indicating each 1-point increase in HADS-A and HADS-D corresponded to 3.9% and 6.2% higher DT scores, respectively. Patient disease stage significantly impacted caregiver DT score, with those caring for stage IV patients showing higher distress compared to stage 0 (p = 0.002), Similarly elevated distress was observed for caregivers of stage III (p = 0.007) and stages I/II patients (p ≤ 0.001) when compared to the stage 0. Notably, patient demographics (age, sex) and caregiving duration showed no significant associations (Table 6).

**Table 6:** Multivariable generalized linear model for caregiver DT score.

Variable	Adjusted Risk Ratio (RR)	95% CI for RR		p-value
		**Lower**	**Upper**	
**(Intercept)**	1.168	0.677	2.013	0.577
Caregiver age (years)	1.002	0.996	1.009	0.466
Caregiver sex (Male vs Female)	0.820	0.712	0.945	0.006
Patient sex	0.965	0.822	1.132	0.659
Patient age (years)	1.003	0.997	1.008	0.335
Duration as caregiver (years)	1.015	0.942	1.094	0.696
Caregiver anxiety score (HADS-A)	1.039	1.018	1.060	<0.001
Caregiver depression score (HADS-D)	1.062	1.039	1.086	<0.001
**Cancer Stage (Reference: Stage 0)**				
Stage 4	1.782	1.247	2.546	0.002
Stage 3	1.651	1.150	2.372	0.007
Stage 2	1.840	1.270	2.664	0.001
Stage 1	2.101	1.385	3.188	<0.001

## Discussion

Our study further demonstrates the clinical utility of the DT as an efficient and user-friendly tool for identifying psychological distress in caregivers. Unlike more complex assessment instruments, the DT offers a practical solution for busy oncology settings where time constraints often limit comprehensive psychological evaluations, while still effectively highlighting caregivers in need of intervention. This practical advantage is consistent with prior DT validation studies across diverse caregiver populations, where brevity and ease of administration were identified as key strengths facilitating routine clinical use [13–15]. Furthermore, the mean DT score in our study was 3.6 (SD = 2.797), consistent with several findings in the literature. Studies in similar settings reported comparable mean DT scores, such as 3.61 (SD = 2.26) for caregivers of Chinese breast cancer patients receiving chemotherapy and 3.67 in a study validating DT use in cancer patients’ families [5,12]. However, higher mean DT scores were observed in studies involving caregivers during more intensive treatment phases, such as combined chemoradiotherapy (mean = 5.2, SD = 2.5) and radiotherapy (mean = 6.46, SD = 2.43) [22,23]. These variations emphasize the impact of treatment intensity on caregiver distress and highlight the importance of targeted interventions in higher-stress contexts.

By establishing a validated cutoff score and confirming the DT’s reliability in detecting anxiety and depression, this study underscores its practical application in routine clinical practice. Our study established a DT cutoff score of 5.5 for anxiety and 6.5 for depression. Using the HADS cutoff score of 8 for each of the subscales as the standard, the ROC analysis demonstrated an AUC of 0.816 (95% CI = 0.759–0.872; p < 0.001) for anxiety and 0.818 (95% CI = 0.761–0.876; p < 0.001) for depression. This indicates that DT is an effective screening tool for anxiety and depression. While our cutoffs are higher than those reported in some comparable studies (e.g., cutoffs of 4/5 or 5) [5,12], similar variability in optimal DT thresholds has been documented across caregiver populations and cultural contexts, with reported cutoffs of 4 for anxiety and 5 for depression in caregivers of persons with multiple sclerosis [14], a cutoff of 4 in parents caring for chronically ill children [15], and higher cutoffs of up to 7 in caregivers of children and adolescents with schizophrenia [16].

These differences likely reflect population variations, as caregivers may experience elevated baseline distress compared to patients and family members. Furthermore, cultural differences in distress reporting, along with the advanced disease stages of patients in our sample (predominantly stages III–IV), may have contributed to generally higher distress levels among caregivers—potentially shifting the optimal discrimination thresholds upward. Despite such variations in cutoff scores, studies of the DT across caregiver populations—including caregivers of persons with multiple sclerosis and parents of chronically ill children—have consistently demonstrated strong discriminant validity with acceptable AUC values [14,15]. Importantly, across studies, including ours, the DT demonstrated excellent discriminant performance, with AUC values ranging from 0.81 to 0.85 across caregiver populations of cancer patients [5]. This consistency strengthens confidence in the DT as a reliable screening tool for psychological distress in caregivers of cancer patients.

Our analysis of factors influencing distress yielded several key insights. As expected, a strong positive correlation was found between caregivers’ total HADS scores and their DT scores, a finding consistent with prior validation work [5]. Furthermore, significant gender differences emerged, with female caregivers reporting markedly higher distress levels than males, a result aligning with studies on caregiver quality of life [24], potentially due to societal expectations and caregiving roles.

Additionally, the stage of a patient’s disease had a significant effect on caregiver distress levels. Caregivers of stage IV patients reported greater distress than those caring for stage 0 patients. Similarly, caregivers of stage III patients and those caring for patients with stage I/II disease also experienced higher distress compared to stage 0 caregivers. These results are likely due to increased caregiving demands and prognostic uncertainty. This aligns with studies identifying disease severity as a predictor of caregiver distress [4], though some variability exists in the literature [22]. Notably, caregiver age and caregiving duration were not significantly correlated with DT scores in our sample, a finding supported by some studies [24] though contrary to others that suggest younger caregivers may be more vulnerable [4]. Moreover, both HADS anxiety and depression scores were significant predictors of caregivers’ DT scores, with each one-point increase in anxiety associated with a 3.9% rise in DT score, and each one-point increase in depression associated with a 6.2% rise in DT score. These results reaffirm previous findings that distress thermometer scores are reflective of psychological distress, as demonstrated by studies linking DT scores with caregivers’ anxiety and depression symptoms [4,5]. Finally, a significant correlation between patient and caregiver DT scores suggests a dyadic interplay of distress, supporting research that found distress levels in patients and caregivers change in tandem over time [24]. Family caregivers of cancer patients frequently experience depression and anxiety due to the substantial emotional, physical, and financial demands of caregiving. Addressing these mental health challenges is vital for both caregiver well-being and the quality of patient care.

In this study, 12% of caregivers exhibited definite anxiety and 9.8% exhibited definite depression, with an additional 12% and 14.9% displaying borderline anxiety and depression, respectively. These rates exceed the 8.9% prevalence reported among caregivers of Chinese breast cancer patients [12] but remain lower than the 48.6% anxiety and 29.4% depression documented in a Malaysian oncology clinic [25]. Such variations underscore the importance of implementing routine psychological screening tailored to specific caregiver populations. Within culturally diverse settings such as Saudi Arabia—where stigma and social norms may inhibit open disclosure of emotional distress—the DT offers a brief, non-stigmatizing approach that aligns well with caregiver needs and healthcare system constraints [17,18].

## Conclusion

This study has established the validity of the Arabic version of DT as a screening tool for distress among caregivers of cancer patients. The DT demonstrated strong predictive validity, with optimal cutoff scores of ≥5.5 for anxiety and ≥6.5 for depression. Nearly half of caregivers experienced significant distress, with notable prevalence of anxiety and depression symptoms. Caregiver distress was significantly correlated with both anxiety and depression scores, and was further influenced by caregiver gender and the patient’s cancer stage. Further studies are needed to assess the utility of routine psychological screening of caregivers, particularly those supporting patients with advanced-stage cancer to ensure timely intervention and support.

### Recommendations and Limitations

This study highlights the need for routine psychological screening of cancer caregivers, given the high prevalence of distress, anxiety, and depression. The Arabic DT proved to be a valid, efficient tool and should be adopted in clinical settings using locally validated cutoff scores (≥5.5 for anxiety, ≥ 6.5 for depression). Special attention should be given to female caregivers and those supporting patients with advanced-stage cancer, who are at greater risk. However, the study’s single-center design and culturally specific sample may limit generalizability. To strengthen external validity and better inform national care guidelines, future research should include multi-center studies with more diverse populations and incorporate mental health support into routine oncology care.
